# Metformin *versus* glyburide in treatment and control of gestational *diabetes mellitus*: a systematic review with meta-analysis

**DOI:** 10.31744/einstein_journal/2022RW6155

**Published:** 2022-02-02

**Authors:** Marina Martins de Oliveira, Kayan Felipe de Oliveira Andrade, Giovanni Henrique Silva Lima, Thiago Casali Rocha

**Affiliations:** 1 Faculdade de Ciências Médicas e da Saúde de Juiz de Fora Juiz de Fora MG Brazil Faculdade de Ciências Médicas e da Saúde de Juiz de Fora, Juiz de Fora, MG, Brazil.

**Keywords:** Diabetes, gestational, Glyburide/therapeutic use, Metformin/therapeutic use

## Abstract

**Objective:**

To compare the major outcomes of use of metformin and glyburide in treatment of gestational *diabetes mellitus*.

**Methods:**

Studies published in English, in the last 10 years, in the databases MEDLINE^®^, SciELO, LILACS and Cochrane Library were analyzed, and randomized controlled trials were selected. Health Sciences Descriptors were used to compose the search phrase, and the keywords “Gestational diabetes”, “Glyburide”, “Metformin” and their variations were searched in the Medical Subject Headings. PRISMA systematization was used to prepare this review, and a meta-analysis was conducted aiming to mathematically show the results of fasting blood glucose, postprandial blood glucose, birth weight and weight gain during pregnancy after using metformin and glyburide.

**Results:**

The studies evaluated birth weight, neonatal hypoglycemia, mode of delivery, need for intensive care, Apgar score, macrosomia, fasting glucose, postprandial glucose and weight gain during pregnancy. In 60% of studies, there were no statistically significant differences regarding safety and efficacy of administration of metformin and glyburide. Meta-analysis demonstrated the absence of statistical differences between these drugs in fasting blood glucose (p=0.821), postprandial blood glucose (p=0.217) and birth weight (p=0.194). However, significant differences were shown in weight gain during pregnancy (p=0.036).

**Conclusion:**

The methods are effective, but the adverse effects of glyburide are more common; therefore, the use of metformin should be recommended, if in monotherapy.

## INTRODUCTION

The pregnancy state is already defined as a condition of predisposition to diabetes, due to the production of placental enzymes (which act in the degradation of insulin), and hyperglycemic hormones. This increases their production and tissue resistance, which can evolve to pancreatic cell dysfunction.^(1)^ One of the consequences is gestational *diabetes mellitus* (GDM), which is a carbohydrate intolerance of variable severity. This disease has no previous diagnosis, starting during gestation and possibly leading to risks for the mother, fetus, and newborn. The condition is usually diagnosed in the second or third trimester of pregnancy.^(2)^

Worldwide, GDM is one of the most frequent medical complications of pregnancy, affecting 1% to 35% of pregnant women, depending on the population and the diagnostic criteria used.^(3)^Brazil has a heterogeneous estimate of the population frequency of hyperglycemia during pregnancy, with an estimated prevalence of 18% of GDM in the Brazilian Public Health System (SUS - *Sistema Único de Saúde*).^(4)^

With early diagnosis and correct treatment, maternal and fetal harm and consequences are substantially reduced.^(5)^ Thus, the initial treatment is based on lifestyle changes, but when these are not enough to control glycemic levels alone, drug treatment is required.^(6)^

Insulin is recommended as first line of treatment for GDM by the American Diabetes Association (ADA). In addition, the American College of Obstetricians and Gynecologists (ACOG) considers the efficacy of oral hypoglycemic agents equivalent when compared to insulin, although insulin is used as first line, since there are no studies demonstrating the long-term effects of hypoglycemic agents in pregnancy.^(7)^

The use of glyburide in combination with metformin is already well established for treating type 2 diabetes in non-pregnant women. Such an association may be a desirable approach for women with GDM with glucose levels that remain above the range despite the maximum tolerated by oral monotherapy.^(8)^ This treatment has the potential to avoid discomfort of subcutaneous injections and the high costs of insulin therapy, as well as possible drawbacks, such as doubts about the correct form of use, forgetting the schedule, and even difficulty in accepting the use of insulin, considering it an aggression to the body.^(9,10)^

## OBJECTIVE

To compare the main outcomes of using metformin and glyburide in the treatment of gestational *diabetes mellitus*.

## METHODS

The most relevant studies originally published in English over the last 10 years (January 2010 to May 2020) were analyzed, with reference to the databases National Library of Medicine (MEDLINE^®^), Scientific Electronic Library Online (SciELO), Latin American and Caribbean Health Sciences Literature (LILACS), and Cochrane Library. Aiming to select studies with greater clinical relevance and scientific evidence, only randomized controlled trials (RCT) were included. In preparation of the search phase, this study used the Health Science Descriptors (DeCS), and the following keywords were found: “Gestational diabetes”, “Glyburide”, “Metformin.” The Medical Subject Headings (MeSH) were consulted to identify the variants of the keywords presented above. To prepare this review, the Preferred Reporting Items for Systematic Reviews and Meta-analyses (PRISMA) systematization was used.^(11)^Table 1 shows the exclusion and inclusion criteria adopted.

**Table 1:** Exclusion and inclusion criteria and key endpoints

Inclusion criteria	
Delimitation	Randomized controlled clinical trial
Patients	Women aged over 18 years with gestational age between 11-36 weeks diagnosed with GDM who have failed to control their blood glucose with lifestyle change measures
Intervention	Oral monotherapy: metformin
Control	Oral monotherapy: glyburide
Languages	In English only

**Exclusion criteria**	

Delimitation	Unclear randomization process
Patients	Women with pre-DMG or first-trimester fasting glucose ≥105mg/dL, suspected intrauterine growth restriction before 24 weeks, and major fetal malformations
Intervention	Unclear or inappropriate interventions Need to combine other control intervention (adding another hypoglycemic agent and/or insulin)

**Fundamental clinical outcomes**	

Maternal	Mode of delivery* Pregnancy weight gain* Fasting blood glucose* Postprandial blood glucose*
Neonatal	Birth weight* Macrosomia* Neonatal hypoglycemia^†^ Apgar score^†^ Intensive care^†^

* Outcomes related to efficacy of treatment; ^†^ outcomes related to drug safety.

GDM: gestational *diabetes mellitus*.

### Statistical analysis

A meta-analysis of data on fasting blood glucose, postprandial blood glucose, gestational weight gain, and birth weight gain was performed using MedCalc 15.8 software, using a fixed-effect and random-effect statistical analysis, taking into consideration heterogeneity of the studies. The 95% confidence interval (95%CI) was calculated exclusively for each study, followed by the calculation related to the combination of selected studies. The mean and standard deviation of each study were checked and only p-values <0.05 were adopted as significant.

## RESULTS

**Figure 1:**
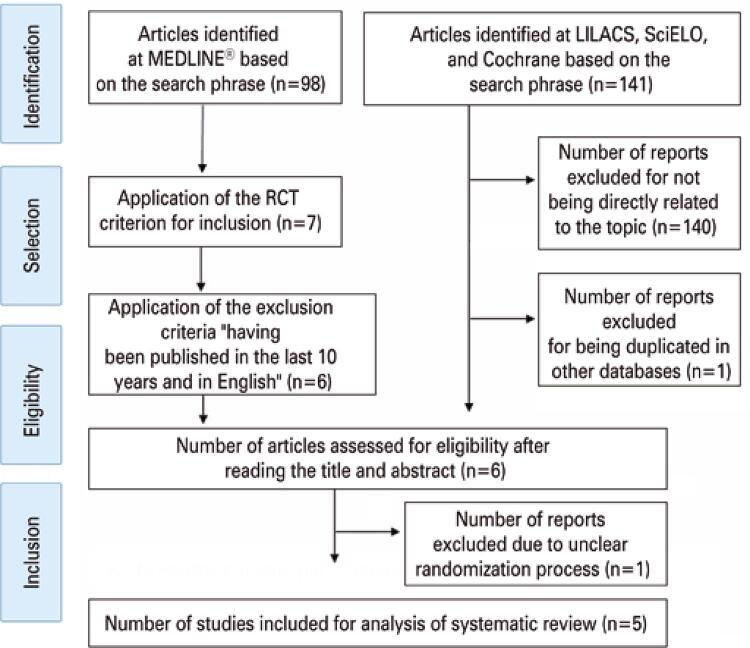
Selection of articles for the review

LILACS: Latin American and Caribbean Health Sciences Literature; SciELO: Scientific Electronic Library Online; RCT: randomized controlled trial.

A total of 239 studies involving GDM, glyburide, and metformin were identified. However, from the application of the previously defined criteria, only five were part of the scope of this review (Figure 1). Thus, for the purpose of inclusion in this review, only RCTs were considered.

The studies reviewed involved 684 pregnant patients from 11 to 36 weeks of gestation. Among the selected RCTs, three concluded, when comparing safety and efficacy of metformin and glyburide administration, there were no significant differences.^(12-14)^

However, as shown on table 2, there is evidence that neonatal complications are significantly milder and less common in newborns of women treated with metformin. Additionally, weight gain during pregnancy is also lower with metformin compared to the use of glyburide.^(12-15)^

**Table 2:** Characteristics of the main studies and their results for the use of glyburide and metformin in cases of gestational *diabetes mellitus*

Study	Patients	Intervention and methods	Maternal and neonatal outcomes								

			Birth weight	Neonatal hypoglycemia	Need for Caesarean section	Need for intensive care	Apgar score	Macrosomia	↓ Fasting blood glucose	↓ Post-prandial blood glucose	Weight gain in pregnancy
Nachum et al.^(12)^	104 women 53 GG 51 GM	GG: 2.5-20mg/day GM: 850-2,550mg/ day after meals	p=0.6 M=G*	p=0.09 M>G^†^	p=0.7 M=G*		Apgar score>7 p=1 M=G*	>4,000g p=0.6 M=G*	p=0.2 M=G*	p=0.3 M=G*	p=0.8 M=G*
Silva et al.^(13)^	200 women 96 GG 104 GM	GG: 5-20mg/day GM: 1,000-2,500mg/day	p=0.01 M>G^†^	p=0.81 M=G*	p=0.88 M=G*	p=0.94 M=G*	In 1’: p=0.56 Up to 5’: p=0.50 M=G*		p=0.18 M=G*	p=0.98 M=G*	p=0.04 M>G^†^
Silva et al.^(14)^	72 women 40 GG 32 GM	GG: 5-20mg/day GM: 500-2,500mg/day	p=0.36 M=G*	p=0.89 M=G*	p=0.91 M=G*	p=0.23 M=G*	In 1’: p=0.57 In 5’: p=0.24 M=G*	>4kg p=0.24 M=G*	p=0.15 M=G*	p=0.10 M=G*	p=0.02 M>G^†^
George et al.^(15)^	159 women 80 GG 79 GM	GG: 2.5-15mg/day GM: 500-2,500mg/day		p=0.001 M>G^†^				>3.7kg p=0.73 M=G*	p=0.37 M=G*	p=0.28 M=G*	
Moore et al.^(16)^	149 women 74 GG 75 GM	GG: 5-20mg/day GM: 500mg-2g/day	p=0.02 M>G^†^	p=0.32 M=G*	p=0.02 G>M^‡^	p=0.37 M=G*			p=0.23 M=G*	p=0.24 M=G*	

GG: Glyburide Group; MG: Metformin Group;

* No significant difference between the use of metformin and glyburide at outcome; ^†^ metformin was superior to glyburide at outcome; ‡ glyburide was superior to metformin at outcome.

### Meta-analysis

The five studies in this review provide satisfactory information to analyze fasting glucose, postprandial glucose, and neonatal weight in the Glyburide Group *versus* the Metformin Group,^(12-16)^ and provide data to compare maternal weight gain in three studies.^(12-14)^

Based on the five articles mentioned, with a sample of 684 volunteers, a meta-analysis was performed regarding fasting blood glucose. For fixed and random effects, respectively, the standardized mean difference values were -0.102 and -0.0365.

**Figure 2:**
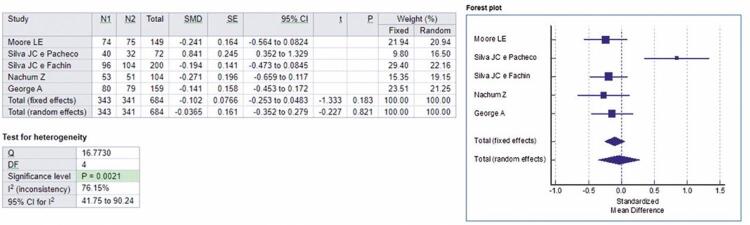
Forest-plot of the studies included in the analysis of the fixed and random effect and standardized mean difference for fasting blood glucose, adopting a 95% confidence interval

N1: Metformin Group sample; N2: Glyburide Group sample; SMD: standardized mean differences; SE: standard error; 95%IC: 95% confidence interval; *t*: *t*-statistic; p: p value; Q: Cochran’s Q statistics; DF: degrees of freedom.

Analysis of the comparative effect of metformin and glyburide on fasting blood glucose in patients with GDM is shown in forest-plot in figure 2, and the absence of statistical differences between these drugs on fasting blood glucose is indicated. Heterogeneity among studies was statistically demonstrated by performing the test of heterogeneity in the analysis, which was significant (p=0.0021). All studies included in this meta-analysis investigated the possible effects of glyburide when treating GDM in doses of 2.5mg to 20mg per day, *versus* metformin in doses of 500mg to 2,550mg per day.

Four of the five studies included in this review provided sufficient data to analyze postprandial glycemia in Glyburide Group *versus* the Metformin Group.^(12-16)^Based on the four articles mentioned, with a total sample of 525 volunteers, a meta-analysis was performed. For fixed and random effects, respectively, the standardized mean difference values were -0.102 and -0.0365.

**Figure 3:**
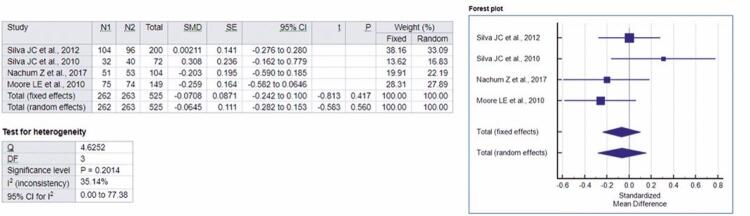
Forest-plot of the studies included in the analysis of the fixed random effect and standardized mean difference concerning postprandial glycemia, adopting a 95% confidence interval

N1: Metformin Group sample; N2: Glyburide Group sample; SMD: standardized mean differences; SE: standard error; 95%IC: 95% confidence interval; *t*: *t*-statistic; p: p value; Q: Cochran’s Q statistics; DF: degrees of freedom.

In figure 3, by forest-plot, the analysis of the effect of metformin and glyburide on postprandial glycemia in patients with GDM is evident, indicating no statistical differences between these drugs in postprandial glycemia. Homogeneity among the studies was also statistically evident by using the heterogeneity test in the analysis, which was not significant (p=0.2014). By the studies included in this meta-analysis, the possibility of effects of glyburide in doses from 2.5mg to 20mg per day, *versus* metformin in doses from 500mg to 2,550mg per day in the treatment of GDM was investigated.

To analyze the gestational weight gain in the Glyburide *versus* Metformin Group, three of the five studies in this review provided sufficient data.^(12-14)^A meta-analysis was performed based on the three articles mentioned, which totaled up 367 volunteers in the sample. The standardized mean difference values were -0.217 and -0.217, respectively, for the fixed and random effects.

**Figure 4:**
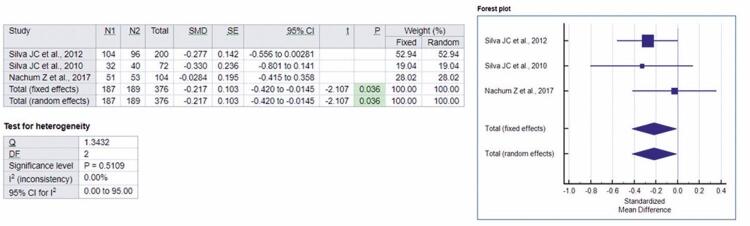
Forest-plot of the studies included in the analysis of the fixed and random effect and standardized mean difference for weight gain, adopting a 95% confidence interval

N1: Metformin Group sample; N2: Glyburide Group sample; SMD: standardized mean differences; SE: standard error; 95%IC: 95% confidence interval; *t*: *t*-statistic; p: p value; Q: Cochran’s Q statistics; DF: degrees of freedom.

Thus, in forest-plot, figure 4 shows the analysis of the effect of metformin and glyburide on weight gain during pregnancy in patients with GDM, indicating the superiority of metformin over glyburide. The presence of homogeneity among the studies was statistically confirmed by applying a heterogeneity test in the analysis, which was not significant (p=0.5109). The possibility of effects of glyburide was analyzed by the studies in doses of 2.5mg to 20mg per day, *versus* metformin in doses of 500mg to 2,550mg per day in the treatment of GDM.

Finally, satisfactory data were provided to analyze birth weight in the Glyburide Group *versus* the Metformin Group by the five studies contained in this review.^(12-16)^ A meta-analysis was performed based on the five articles mentioned, totaling up a sample of 684 volunteers. Respectively, for fixed and random effects, the standardized mean differences were -0.182 and -0.167.

**Figure 5:**
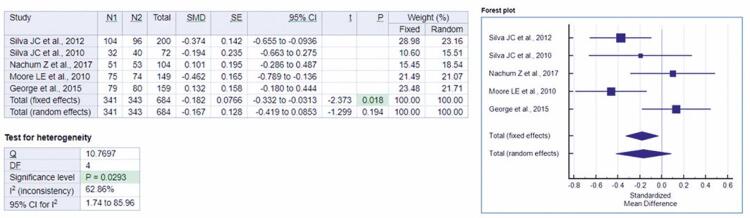
Forest-plot of the studies in the analysis of the fixed and random effect and standardized mean difference for birth weight, adopting 95% confidence interval

N1: Metformin Group sample; N2: Glyburide Group sample; SMD: standardized mean differences; SE: standard error; 95%IC: 95% confidence interval; *t*: t-statistic; p: p value; Q: Cochran’s Q statistics; DF: degrees of freedom.

Figure 5, in a forest-plot, shows the analysis of the effect of metformin and glyburide on birth weight in patients with GDM, indicating no statistical differences between these drugs on weight gain. A significant value (p=0.0293) was presented for the heterogeneity test applied in the analysis, which statistically highlights heterogeneity among the studies. The studies included in this meta-analysis investigated the possibility of effects of glyburide in doses of 2.5mg to 20mg per day, *versus* metformin in doses of 500mg to 2,550mg per day in the treatment of GDM.

## DISCUSSION

Our results confirm the assumption there are no significant differences in terms of safety and efficacy in the administration of metformin and glyburide,^(13,14,16)^ especially in the reduction of fasting glycemia, postprandial glycemia, and birth weight. However, Nachum et al.,^(12)^Silva et al.,^(13)^George et al.,^(15)^and Moore et al.,^(16)^showed greater benefits of metformin when compared to glyburide, in terms of neonatal complications and weight gain during pregnancy; the latter result was evident in our meta-analysis. It was also noted other studies compared several outcomes, such as pregnancy-induced hypertension, hypertensive disorders, and preeclampsia; but compared only metformin and insulin, or glyburide and insulin, whereas this study compared metformin and glyburide.^(2,17,18)^ This study assessed outcomes different from those of other reviews, such as the need for neonatal intensive care, and was based on more current RCTs.

The studies showed the use of metformin caused lower neonatal weights, due to its ability to decrease insulin concentration by crossing the placental barrier.^(13-16)^ Glyburide, on the other hand, caused increased birth weight, although it did not show higher numbers of newborns with macrosomia.^(12,14-16)^ Furthermore, weight gain during pregnancy was lower in the groups that used metformin when compared to the Glyburide Group, due to the drug action.^(12,14)^

Moore et al.,^(16)^ demonstrated that glycemic control failure in the Metformin Group was 2.1 times higher than in the Glyburide Group, with 34.7% of patients requiring insulin therapy in the former group compared to approximately 16% in the latter. In monotherapy, glyburide was inferior in comparison with metformin monotherapy, insulin monotherapy, association between metformin and glyburide, and association between insulin and metformin - the latter being the most therapeutically successful.^(12,13)^

The pharmacokinetics of metformin is not altered during pregnancy, while the oral clearance rates of glyburide increase.^(14)^ Also, glyburide crosses the placental barrier, reaching 50% to 70% of total plasma concentration, which may account for the fact that relative to metformin, this drug presented a higher risk of neonatal hypoglycemia and neonatal disease (such as respiratory distress and birth injury).^(8,12,13)^

In terms of the combination of metformin, glyburide, and insulin, Reynolds et al.,^(8)^ highlighted the combination of glyburide with metformin appears inferior to the combination of insulin with metformin, due to preliminary data suggesting the latter association provides superior glycemic control, with a lower incidence of glucose excursions to levels below 63.063mg/dL. Other systematic reviews with meta-analysis^(2,17,18)^ demonstrated metformin has been found to be superior to insulin due to lower maternal weight gain, lower rates of gestational hypertension, and neonatal hypoglycemia, macrosomia, and lower postprandial glycemia. Relative to insulin, glyburide had an increased risk of neonatal illness, respiratory distress, neonatal hypoglycemia, birth injury, increased birth weight, and macrosomia. When compared to metformin, glyburide was associated with greater maternal weight gain, birth weight, macrosomia, and neonatal hypoglycemia. These findings lead to the assumption that glyburide should be avoided in the treatment of GDM when metformin and insulin are available.^(2,17,18)^ Among the possible outcomes of using both oral drugs are treatment failure, in which it is necessary to switch to insulin therapy, a change in the pharmacokinetics of the drug, the occurrence of general diseases in neonates, and maternal morbidity.^(12,16)^

Strict control of GDM has traditionally been achieved through intensive insulin therapy.^(7)^ However, it is difficult for pregnant women to comply with this type of therapy, which often requires up to four injections a day, and hinders the treatment and its efficacy, in addition to its high cost. This scenario must be considered in the context of developing countries, such as Brazil. In addition, hypoglycemia can be a problem in intensive insulin therapy.^(13)^

Thus, oral therapeutic agents are better tolerated and may be increasingly used in pregnancy.^(19,20)^ Hence some studies reported combination or monotherapy with glyburide and metformin are good alternative strategies in insulin therapy for control and treatment of GDM.^(2,17,21)^ There is still little knowledge regarding the artifices involved in this greater effectiveness and quality, probably due to a multifactorial aspect. Nachum et al.,^(12)^ stated the combination of these drugs showed high efficacy rates with significantly reduced need for insulin, which leads to support the benefit of using an additional oral hypoglycemic agent in case of failure of the first monotherapy treatment before switching to insulin, which should be reserved only for patients who do not respond to both oral treatments, or who have side effects with both oral hypoglycemic agents.

This study had difficulties/limitations during its conduction, especially regarding the small number of studies directly related to monotherapy with metformin or glyburide, making the sample space for analysis small. It is worth noting that no data on long-term effects of these drugs were found in the literature.

## CONCLUSION

There was no difference regarding safety and efficacy of metformin and glyburide administration, particularly on fasting blood glucose, postprandial blood glucose, and birth weight. However, neonatal complications (such as hypoglycemia and breathing difficulties), and weight gain during pregnancy are significantly lower and less common in newborns of women treated with metformin. It is worth noting the need for more randomized controlled trials with large sample sizes, comparing the different treatment strategies (insulin, glyburide, metformin, and their associations). In addition, there is also a need for studies that analyze the long-term effect of these drugs and their comparison.
